# A highly efficacious pediculicide based on dimeticone: Randomized observer blinded comparative trial

**DOI:** 10.1186/1471-2334-8-115

**Published:** 2008-09-10

**Authors:** Jorg Heukelbach, Daniel Pilger, Fabíola A Oliveira, Adak Khakban, Liana Ariza, Hermann Feldmeier

**Affiliations:** 1 Department of Community Health, School of Medicine, Federal University of Ceará, Fortaleza, Brazil; 2 Anton Breinl Centre for Public Health and Tropical Medicine, School of Public Health, Tropical Medicine and Rehabilitation Sciences, James Cook University, Townsville, Australia; 3 Charité University of Medicine, Campus Benjamin Franklin, Institute for Microbiology and Hygiene, Berlin, Germany; 4 Post-Graduation Program in Medical Sciences, School of Medicine, Federal University of Ceará, Fortaleza, Brazil

## Abstract

**Background:**

Infestation with the human head louse (*Pediculus humanus capitis*) occurs worldwide. Existing treatment options are limited, and reports of resistance to commonly used pediculicides have been increasing. In this trial we assessed the efficacy of a product containing a high (92%) concentration of the silicone oil dimeticone (identical in composition to NYDA^®^), as compared to a 1% permethrin lotion.

**Methods:**

Randomized, controlled, observer blinded clinical trial. Participants were recruited from a poor urban neighbourhood in Brazil where pediculosis capitis was highly prevalent. To minimize reinfestation during the trial, participants (145 children aged 5–15 years with head lice infestations) were transferred to a holiday resort outside the endemic area for a period of 9 days. Two applications of dimeticone or 1% permethrin were done, seven days apart. Outcome measures were defined as cure (absence of vital head lice) after first application and before and after second applications, degree of itching, cosmetic acceptability, and clinical pathology.

**Results:**

Overall cure rates were: day 2 – dimeticone 94.5% (95% CI: 86.6% – 98.5%) and permethrin 66.7% (95% CI: 54.6% – 77.3%; p < 0.0001); day 7 – dimeticone 64.4% (95% CI: 53.3% – 75.3%) and permethrin 59.7% (95% CI: 47.5% – 71.1%; p = 0.5); day 9 – dimeticone 97.2% (95% CI: 90.3% – 99.7%) and permethrin 67.6% (95% CI: 55.4%-78.2%); p < 0.0001). Itching was reduced similarly in both groups. Cosmetic acceptability was significantly better in the dimeticone group as compared to the permethrin group (p = 0.01). Two mild product-related incidents occurred in the dimeticone group.

**Conclusion:**

The dimeticone product is a safe and highly efficacious pediculicide. Due to its physical mode of action (interruption of the lice's oxygen supply of the central nervous system), development of resistance is unlikely.

**Trial registration:**

Current Controlled Trials ISRCTN15117709.

## Background

Infestation with the human head louse (*Pediculus humanus capitis*) occurs worldwide, and pediculosis capitis is hyperendemic in many resource-poor populations in the developing world [1,2]. In high income countries, head lice are a problem mainly in school-aged children [3,4], with an increasing number of reports of resistance to commonly used pediculicides, such as permethrin and malathion [5-13]. In addition, there is a growing public concern about the potential hazards of pediculicides with a neurotoxic mode of action. Thus, existing treatment options are limited.

Dimeticones (linear polydimethylsiloxanes of varying chain length) are silicone oils with a low surface tension and special creeping and spreading properties. They are a new class of anti-head lice compounds with a physical mode of action. It has recently been demonstrated that NYDA^®^ (Pohl-Boskamp GmbH & Co. KG, Hohenlockstedt, Germany), an anti-head lice product containing two dimeticones with different viscosities in a total concentration of 92%, rapidly penetrated into the spiracles of lice. The product filled the entire tracheal system within minutes, thereby interrupting oxygen supply and leading to rapid death of the insect [14,15]. In addition, we recently reported a high *in vitro *efficacy of NYDA^® ^against adult head lice [16]. The product is commercialized in Germany and other European countries since 2006 and sold as an over-the-counter medical device.

Two randomized controlled trials in the United Kingdom showed that a product containing only 4% dimeticone (Hedrin^®^) had a similar efficacy as d-phenotrin (0.5%), with cure rates of about 70% [17], and a better efficacy than malathion [18].

In the present observer blinded comparative trial we assessed the efficacy of a product identical in composition with NYDA^® ^in comparison with 1% permethrin lotion (Kwell^®^), in individuals recruited from an area where pediculosis capitis is hyperendemic.

## Methods

### Participants, setting and eligibility criteria

Participants were recruited from a resource-poor community in Fortaleza, the capital of Ceará State in northeast Brazil. In the community, head lice infestations are very common (prevalence in the general population > 40%). In general, intensity of the infestation is high, and pediculosis capitis is associated with considerable morbidity [1]. Children aged 5 – 15 years with a head lice infestation were identified by community health care workers. Individuals were included in the study if one or more active head lice were found by visual inspection. Visual inspections were done by a trained auxiliary nurse, following a standardized procedure. It took three minutes, or was interrupted earlier when 25 lice were found. Fine tooth combing was not performed as a diagnostic test at this stage, as combing would have reduced the number of head lice present on the scalp and thereby bias cure rates.

We opted for a comparatively insensitive diagnostic method (visual inspection) [19,20] to reduce the number of individuals included with only few head lice on their scalp and to select participants with a moderate or a high intensity of infestation.

Individuals were not admitted to the study if one or more of the following criteria were present:

- use of head lice products, anthelminthics, or antibiotics within the previous four weeks;

- severe skin disorders of the scalp (such as generalized impetigo, eczema, psoriasis or chronic dermatitis of unknown origin);

- bleached or colour treated hair within the previous four weeks;

- known sensitivity to any ingredients in the products;

- mental disease;

- drug abuse;

- pregnant or lactating girls;

- unwillingness to stay for 9 days in a holiday resort outside the endemic area where the clinical trial would be carried out;

- participation in another clinical study in the previous month.

### Study location

As recent trials in the United Kingdom have shown rapid reinfestation after cure [17,18] and because reinfestation is known to occur very rapidly in the setting where participants were recruited from (Pilger et al., manuscript submitted), participants were transferred to a holiday resort outside the endemic area for a period of nine days. It was expected that this would reduce the occurrence of re-infestation.

### Intervention

Participants with head lice were randomized to receive either topical treatment with a product containing a high percentage of dimeticones (92%), equivalent in composition to NYDA^® ^(G. Pohl-Boskamp GmbH & Co. KG, Hohenlockstedt, Germany), or permethrin 1% lotion (Kwell^®^, GlaxoSmithKline, Brazil). The first product was prepared at the Department of Pharmacy of the Federal University of Ceará by an experienced pharmacist, the latter bought locally at a pharmacy. As NYDA^® ^was not registered as a medical device in Brazil, a parallel formulation was produced locally. The product was prepared using the identical constituents obtained from the same commercial source as the components of the branded product NYDA^®^. It was stored in 500 ml glass bottles and kept in a refrigerator until use. Permethrin is commonly used in Brazil and many other countries as a first line therapy for head lice infestation. In the study area, permethrin resistance has not been observed. However, there are no systematic studies available, and the resistance situation is not fully understood (Heukelbach, unpublished observation).

Participants were treated immediately upon arrival at the resort (day 1) and, according to the suggestion of a Cochrane Expert Panel [21], a second time 7 days later (day 8) to kill newly hatched lice from eggs which may have survived the first treatment. The producers of both products claim that the substances have an ovicidal effect.

The products were used according to the producers' recommendations. However, following a suggestion made by Dodd [21], the fine tooth comb provided by both producers together with the pediculicide was not used after the application of the products. The dimeticone-based product was applied to dry hair and then left to dry naturally. After 8 hours the hair was washed with a commercial shampoo not containing dimeticones. Permethrin was applied to wet hair, left for 30 minutes and thereafter washed out in an identical manner as dimeticone.

Both products were applied systematically onto the hair from the hair shafts to the tips, and a normal comb was used to spread the liquids evenly.

### Objective

The trial was done to test the efficacy of a dimeticone-based product chemically identical to NYDA^® ^to cure head lice infestations, in comparison to permethrin 1% lotion (Kwell^®^).

### Outcomes

The primary outcome measure was defined as the proportion of participants cured of head lice infestation 1, 6 and 8 days after the first treatment (i.e. days 2, 7 and 9, respectively).

Cure was defined as the complete absence of viable lice on the scalp, as determined by wet combing with a high quality plastic head louse comb. This is considered a sensitive method to diagnose active lice infestation [19]. Diagnostic wet combing was performed after the application of a commercially available conditioner without silicone oil. Prior to the study, the conditioner had been tested in vitro to exclude any pediculicidal effect (20 head lice were fully coated with the conditioner, placed on a humid paper and observed for vital signs). Diagnostic wet combing was performed on day 2 (24 hours after first application), day 7 (one day before the second treatment) and day 9 (24 hours after second treatment). Head lice found were carefully removed and examined after 0.5 to 2 hours under a dissecting microscope for vitality signs as described previously, and stringent criteria for mortality were used [16].

**Figure 1:**
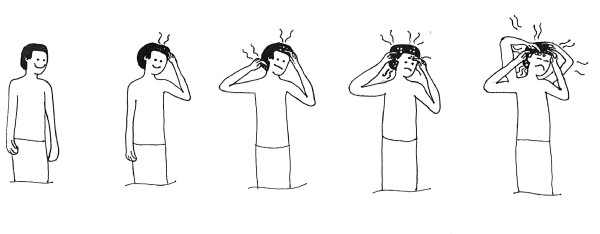
Assessment of degree of itching using an ordinal visual scale ranging from 0 to 4.

Secondary outcomes included the reduction of clinical pathology, reduction of the degree of itching, cosmetic acceptability of the products, and safety (number and type of adverse events). The degree of itching was assessed daily based on a pre-tested ordinal visual analogue scale ranging from 0 to 4 (Figure 1). Cosmetic acceptability was assessed using a summary score ranging from -4 (extremely negative) to +4 (extremely positive), using a standardized questionnaire including subjective assessment of smelling, irritation of scalp, cosmetic changes of hair, and changes in the easiness to comb the hair. Clinical pathology included the presence of erythema, papules, excoriations, eczema, secondary infection and enlarged cervical or retro-auricular lymph nodes.

The assessment of clinical pathology was made before each application (days 1 and 9) and on days 2, 4 and 7. Cosmetic acceptability was determined on days 2, 4, 7 and 9.

Assessors for cure rate, itching, clinical pathology, and cosmetic acceptability were blinded regarding treatment groups.

Participants were instructed to report any adverse events at once and were specifically asked about possible adverse events at assessments of cosmetic acceptability. All adverse events reported by participants or noted by investigators during examination, and any inter-current illness was recorded in an Adverse Event Report Form, regardless whether or not they were considered to be related to the intervention.

The final assessment was undertaken 24 hours after the second treatment. If lice were found, they were removed and collected for analysis of activity. Before leaving the holiday resort, participants were treated with ivermectin 200 μg/kg in a single dose. In Brazil, oral ivermectin is a registered treatment for pediculosis.

### Sample size

Assuming a cure rate of 80%, a study size of 62 participants would be needed per group to detect an absolute difference of 25% in cure rates between treatment groups (power = 80%). Considering loss to follow-up (children who wanted to return from the holiday resort to their homes during the study period) and possible post-randomization protocol violations, a study size of about 70 participants per group was considered to be sufficient.

### Random allocation

Participants had an equal probability of assignment to the groups. The randomization code was created by an investigator not involved in assessment of outcome measures or analysis of the study, using a computer generated random list. Blocked randomisation was used with a block length of six.

### Blinding

The study was observer-blinded. As the two products looked considerably different, had a different smell and distinct cosmetic characteristics, a double-blinded design was not possible. All study personnel directly or indirectly involved in assessments of primary or secondary outcomes were blinded to treatment assignment for the duration of the study. Only the auxiliary personnel who applied the topical treatment and one investigator supervising the treatment had access to unblinded data. Although complete blinding of the participants was not possible, they were not informed to which treatment groups they belonged to. Treatment and assessment of outcome measures were done at distinct locations of the holiday resort. During the study period each participant carried an ID number in a badge so that no study participant could be mistaken for someone else.

### Statistical methods

Data were entered twice into an Epi Info data base (version 6.04d, CDC, Atlanta, USA) and cross-checked for entry errors. Data analysis was done using STATA software (version 9; Stata Corporation, College Station, USA).

Analysis was based on the intention-to-treat population. The intention-to-treat population included all participants allocated to one treatment group and who received at least the first treatment. Differences between the groups in baseline characteristics, safety, cosmetic acceptability, reduction of clinical pathology and efficacy was tested using the chi squared test, the Fisher's exact test and the Mann-Whitney test, where appropriate.

### Ethical aspects

This study was conducted in accordance with the revised Declaration of Helsinki. Each participant and his/her guardian gave written informed consent after having received an information leaflet and after a verbal explanation of the objectives and the procedures of the study had been given. At the end of the study, all participants were treated with oral ivermectin to cure any persistent head lice infestation, and to eliminate intestinal helminths. Ivermectin is known to be an effective drug against a variety of ectoparasites and intestinal helminths [22,23].

The study was approved by the Ethical Review Board of the Federal University of Ceará.

## Results

### Recruitment

The trial was divided into two parts. Two holiday camps were organized at the same resort in January 2007, each with duration of nine days. Both camps were carried out during school holidays. A total of 145 children aged 5 to 15 years agreed to participate. In the first camp, 77 individuals participated, and in the second 68. Conditions at both camps were identical. Randomization was done only once, before the beginning of the study. There was no difference in demographic or clinical characteristics of study participants at both camps.

### Participant flow (Figure 2)

**Figure 2:**
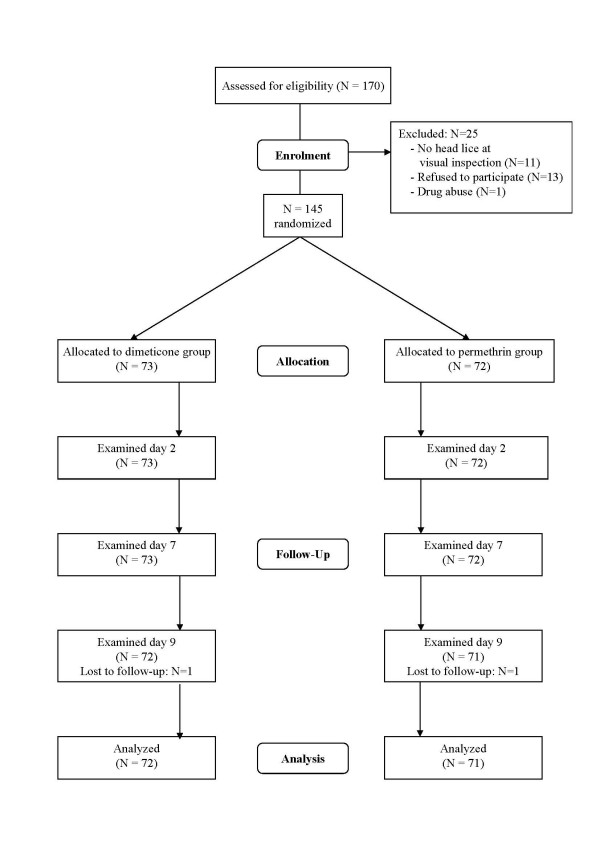
Flow of participants through each stage of the trial.

The total number of participants assigned to the dimeticone group was 73, and to the permethrin group 72. All 145 participants received two treatments. All participants were examined for the primary outcome at days 2 and 7. In each group, one participant was lost during follow-up. Consequently, at day 9, 72 participants in the dimeticone and 71 in the permethrin group were available for assessment of cure.

Eight participants in the dimeticone group and five in the permethrin group left the holiday resort during the study period due to homesickness. They were visited in their homes at the respective days to receive second treatment and to assess primary and secondary outcome measures, and were included in analysis. Their family members were treated with ivermectin to reduce the occurrence of reinfestation.

### Baseline data

Both treatment groups were similar in age, sex, intensity of infestation, and hair length (Table 1).

**Table 1:** Baseline demographic and clinical characteristics of both treatment groups

	Dimeticone group (n = 73)	Permethrin group (n = 72)	P value
Sex:			
Male	54 (74.0%)	55 (76.4%)	
Female	19 (26.0%)	17 (23.6%)	P = 0.7
Age (years):			
Median (interquartile range)	10 (7–12)	10 (8.5–12)	P = 0.2
Intensity of infestation*			
Median (interquartile range)	4 (2–12)	6 (2–14)	P = 0.5
Hair length:			
Short	21 (28.8%)	21 (29.2%)	
Middle-sized	16 (21.9%)	13 (18.1%)	
Long	36 (49.3%)	38 (52.8%)	P = 0.8

*Number of lice found during three minutes of visual inspection. Inspection was stopped when 25 vital lice were found.

### Cure rates

Cure rates in the dimeticone group were very high at days 2 and 9 and significantly better than in the permethrin group. Cure rates at days 2, 7 and 9 are depicted in Table 2.

**Table 2:** Cure rates, defined as the complete absence of active lice.

	**Dimeticone group**		**Permethrin group**		**P value**	**Effect size**
	**Cured/total**	**% (95% CI)**	**Cured/total**	**% (95% CI)**		**RR (95% CI)**
Day 2	69/73	94.5% (86.6% – 98.5%)	48/72	66.7% (54.6% – 77.3%)	P < 0.0001	1.42 (1.19–1.68)
Day 7	47/73	64.4% (53.3% – 75.3%)	43/72	59.7% (47.5% – 71.1%)	P = 0.5	1.22 (0.59–2.52)
Day 9	70/72	97.2% (90.3% – 99.7%)	48/71	67.6% (55.4% – 78.2%)	P < 0.0001	1.44 (1.22–1.70)

Topical treatments were applied on days 1 and 8.

As an indicator for reinfestation, we assessed the presence of adult lice on day 7 in the head louse-free population on day 2. In the dimeticone group, of the 26 participants with head lice on day 7, 24 had been classified as cured on day 2, and only 2 had head lice at both points of time. Of the 24 incident cases, 21 (87.5%) had adult head lice on their scalps on day 7. In the permethrin group, there were 29 participants with head lice on day 7. Of these, 13 were not infested on day 2, and 16 continued being infested. Of the 13 incident cases in this group, 12 (92.3%) had adult head lice on their scalps on day 7.

Participants were stratified according to the number of head lice found at the beginning of the study. Cure rates were high in both treatment groups for participants with low or moderate intensity of infestation, with a significant higher efficacy of dimeticone at day 2 and day 9 (Table 3). In heavy infestation (arbitrarily defined as ≥ 5 active head lice after 3 min visual inspection), the cure rate remained very high in the dimeticone group at days 2 and 9, whereas in the permethrin group it was low.

**Table 3:** Cure rates stratified according to infestation intensity (assessed by 3 minutes of visual inspection before treatment)

	**Dimeticone group**		**Permethrin group**		**P value**	**Effect size**
	**Cured/total**	**% (95% CI)**	**Cured/total**	**% (95% CI)**		**RR (95% CI)**
Low or moderate infestation^a^						
Day 2	37/38	97.4% (86.2% – 99.9%)	23/31	74.2% (55.4% – 88,1%)	P = 0.004	1.31 (1.06–1.63)
Day 7	32/38	84.2% (68.7% – 94.0%)	23/31	74.2% (55.4% – 88,1%)	P = 0.3	1.14 (0.88–1.46)
Day 9	36/37	97.3% (85.5% – 99.9%)	25/31	80.6% (62.5% – 92.5%)	P = 0.02	1.21 (1.01–1.45)
Heavy infestation^b^						
Day 2	32/35	91.4% (76.9% – 98.2%)	25/41	61.0% (44.5% – 75.8%)	P = 0.002	1.50 (1.15–1.95)
Day 7	15/35	42.9% (26.3% – 60.4%)	20/41	48.8% (32.9% – 64.9%)	P = 0.6	0.88 (0.54–1.44)
Day 9	34/35	97.1% (85.1% – 99.9%)	23/40	57,5% (40.9% – 73.0%)	P < 0.0001	1.69 (1.29–2.22)

^a ^< 5 active head lice at the beginning of the study; see material and methods

^b ^≥ 5 active head lice

In the dimeticone group, cure rates were similar at all three assessments when stratified by hair length and sex. Cure rates were also similar in the permethrin group at days 2 and 7, but differed significantly at day 9 with a higher cure rate in participants of male sex (59.3% in females and 94.1% in males; p < 0.01) and with shorter hair (90.5%, 69.2% and 54.1% for short, middle-sized and long hair, respectively, p = 0.02).

### Secondary outcomes

Degree of itching (on an ordinal scale from 0 to 4) was reduced similarly in both treatment groups (Table 4).

**Table 4:** Degree of itching, assessed by an ordinal visual scale from 0 to 4 (see Methods)

	**Dimeticone group**	**Permethrin group**	**P value**
	Median (interquartile range)	Median (interquartile range)	
Before treatment	2 (1–4)	2 (1–3)	p = 0.02
6 h after treatment	1 (0–1)	1 (0–1)	P = 0.8
24 h after treatment (day 2)	1 (0–1)	1 (0–1)	P = 0.6
Day 3	1 (0–1)	1 (0–1)	P = 0.07
Day 4	1 (0–1)	0 (0–1)	P = 0.8
Day 5	1 (0–1)	0 (0–1)	P = 0.13
Day 6	1 (0–1)	0 (0–1)	P = 0.13
Day 7	1 (0–1)	1 (0–1)	P = 0.14
Day 8	1 (0–1)	0 (0–1)	P = 0.3
Day 9	0 (0–1)	0 (0–1)	P = 0.5

Both treatments were perceived as cosmetically pleasant with a significantly better acceptance of dimeticone at days 4, 7 and 9 (Table 5).

**Table 5:** Cosmetic acceptability of products (minimum score: -4; maximum score: +4; see Methods)

	**Dimeticone group**	**Permethrin group**	**P value**
	Median (interquartile range)	Median (interquartile range)	
Day 2	1 (0 – 2)	1 (-1 – 2)	P = 0.5
Day 4	1 (0 – 2)	0 (-0.5 – 1)	P = 0.003
Day 7	1 (1 – 2)	1 (0 – 2)	P = 0.04
Day 9	1 (1 – 2)	1 (-1 – 2)	P = 0.01

Frequency of cervical lymphadenopathy decreased similarly in both groups during the study period (Table 6). Bacterial superinfection was rare and not observed on day 7 or later.

**Table 6:** Clinical pathology in both treatment groups

	**Dimeticone group** n (%)	**Permethrin group** n (%)	**P value**
Cervical lymphadenopathy:			
Before treatment	49/73 (67.1%)	54/72 (75.0%)	P = 0.3
Day 2	51/73 (69.9%)	52/72 (72.2%)	P = 0.8
Day 4	52/73 (71.2%)	49/72 (68.1%)	P = 0.7
Day 7	39/73 (53.4%)	46/72 (63.9%)	P = 0.2
Day 9	41/72 (56.9%)	45/71 (63.4%)	P = 0.7
Bacterial superinfection of lesions:			
Before treatment	1/73 (1.4%)	0/72 (0%)	P = 0.3
Day 2	1/73 (1.4%)	1/72 (1.4%)	P = 1.0
Day 4	1/73 (1.4%)	0/72 (0%)	P = 0.3
Day 7	0/73 (0%)	0/72 (0%)	P = 1.0
Day 9	0/72 (0%)	0/71 (0%)	P = 1.0

### Adverse events

The number of participants experiencing any adverse events was similar in both groups, and only two product-related incidents occurred.

In the dimeticone group, 29 adverse events were reported in 25 participants. Of these, two were related to treatment (ocular irritation). The liquid had entered into the eyes after topical application. The irritation resolved spontaneously in both cases after washing the eyes with clean water. The other events were classified as unrelated or unlikely to the study (such as superficial wounds after falls, headache etc.).

In the permethrin group, 32 adverse events were reported in 26 participants, all of them unrelated or unlikely to be related to treatment.

## Discussion

Our study shows that a product containing a high percentage of dimeticone (a parallel formulation of NYDA^®^) is significantly more effective in curing head lice infestations than permethrin lotion (Kwell^®^). Dimeticone reduced the degree of itching similar to permethrin and had a higher cosmetic acceptability.

In contrast to the high cure rates in the dimeticone group, cure rates in the permethrin group were 67% and 68% at days 2 and 9. Interestingly, in two recent trials from the UK assessing the efficacy of 4% dimeticone, cure rates of about 70% were reported, but in moderately infested individuals, cure rates were only 39% and 64%, respectively [17,18]. In our study, in the dimeticone group cure rates were not influenced by the intensity of infestation, contrary to the permethrin group.

It may be argued that in countries where resistance to permethrin or malathion has been known since long, such as in the UK, a dimeticone product may perform better, simply because a part of the head lice population would be resistant to insecticides. We believe that the difference in efficacy observed between dimeticone and permethrin in our study reflects the failure of permethrin to kill all head lice on the scalp. This resulted in persistence of infestation in the children treated with this pediculicide, particularly when the intensity of infestation was high. However, we are aware that permethrin resistance cannot be ruled out – although it seems unlikely – because resistance of head lice to insecticides has never been studied systematically in northeast Brazil.

Seven days after the first application, cure rates were considerably lower. This observation can be attributed mainly to reinfestation initiated by children who had remained infested after the first treatment: the vast majority of those study participants that were cured on day 2, but infested on day 7, had vital adult lice detected on this day. As adult head lice found up to one week after cure by definition derive from reinfestation (no newly hatched nymphs can develop into adults within one week) [21,24,25], we believe that the increase in the number of infested children seven days after first application is caused mainly by reinfestation and not by a low ovicidal activity of dimeticone. Children played together during the day, frequently had intimate body contact and slept together in dormitories, irrespective to which treatment group they belonged to. Hence, our data not only show the comparative efficacy of the two tested compounds, but also the effectiveness of head lice treatment in the setting of a holiday resort.

Other studies also provided evidence that reinfestation is common if study participants are not completely cured, or if they stay in close contact to other infested people [17,18]. In fact, we observed in another study that NYDA^® ^reduced hatch rates of eggs to < 4% after 60 min incubation, as compared to a hatch rate of 80% in eggs treated with 0.5% permethrin alcoholic solution (Heukelbach, unpublished data). A few children had mixed stages including nymphs (data not shown). As larval stages were not further defined during the study, we were unable to prove that in some cases eggs may have not been killed by the initial treatment.

Dimeticones are regarded as chemically inert and non-toxic, and are components of many cosmetic skin and hair care products. Since the mode of action of dimeticones is physical, development of resistance is unlikely. Besides, the repeated applications are not expected to increase the risk for adverse events. The two randomized clinical trials recently performed in the UK compared dimeticone in a concentration of only 4% (Hedrin^®^) with α-phenotrin and malathion, respectively [17,18]. We opted to assess the therapeutic efficacy of a product containing a 92% mixture of two dimeticones of different viscosity (a low viscosity, also at room temperature more readily volatile dimeticone, responsible for the highly creeping and spreading properties of the product, and a higher viscosity, less volatile dimeticone) in an environment where the intensity of infestation was extremely high. As NYDA^® ^is claimed to kill lice by entering into the spiracles and filling the tracheal system with subsequent blocking of the oxygen supply [14,15], it can be assumed that the high creeping and spreading properties of the product in connection with the high percentage of the more viscous dimeticone component in NYDA^® ^is responsible for the increased cure rates, as compared to Hedrin^®^. In fact, we have shown that NYDA^® ^killed all lice *in vitro*, collected from individuals living in the community where our participants were recruited from, and that it performed better than 4% dimeticone (Hedrin^®^) [16].

Our study is subject to limitations. Usually, a period of 14 days is recommended for the final assessment, to detect all lice hatching from eggs not killed by the products [21,26]. However, we had perceived in discussions with community representatives prior to the study that neither parents nor children would have agreed to a prolongation of the stay in the resort for more than 10 days. Thus, we could not adhere to recommendations made previously and opted to perform the final assessment after nine days.

In the dimeticone group we observed only two adverse events related to the use of the product. The product had entered the eyes and caused mild irritation which resolved quickly. We conclude that the dimeticone-based product is a safe pediculicide. Dimeticones are physiologically inert and non-toxic silicone oils. They are widely used in cosmetic products to facilitate the use of a comb and to make the hair silky and soft [27]. After oral ingestion, the oil is not absorbed but eliminated unaltered in the faeces. Oral dimeticones are used as anti-flatulents to alleviate gastrointestinal discomfort.

Non-insecticidal treatment options against head lice infestations are limited and include some natural products with good efficacy [28,29], but also physical methods and home remedies with rather low efficacy, such as combing with a fine tooth comb or application of vinegar [30-34]. The dimeticone product, being highly efficacious and non-toxic, can be considered an ideal pediculicide. It is acceptable for individuals who do not want to use insecticides with a neurotoxic potential and for those who look for a high cosmetic acceptability. A recent study has shown that time necessary to treat children with head lice infestations is an important aspect for parents to opt for one or another therapy [35]. As efficacy of dimeticone was very high without using a head louse comb, dimeticone will also be ideal for those parents who find combing nasty and time consuming.

## Conclusion

The dimeticone-based head lice product (similar to the branded product NYDA^®^) is an efficacious alternative to chemical pediculicides with no inherent risk for development of resistance. The cure rate was >97%, even in individuals with a high intensity of infestation. Severity of itching was reduced to negligible.

## Competing interests

JH and HF have been scientific consultants to G. Pohl-Boskamp GmbH & Co. KG, the producer of NYDA^®^. The company had no role in the design, execution, or interpretation of the study. The other authors do not have any conflicts of interest to declare.

## Authors' contributions

JH: study design, conducted the study, statistical analysis, contributed to the manuscript; DP: study design, conducted the study, statistical analysis, contributed to the manuscript; FAO: study design, conducted the study, contributed to the manuscript; AK: conducted the study, contributed to the manuscript; LA: conducted the study, statistical analysis, contributed to the manuscript; HF: study design, contributed to the manuscript. All authors read and approved the final manuscript.

## Pre-publication history

The pre-publication history for this paper can be accessed here:


